# A20: a master regulator of arthritis

**DOI:** 10.1186/s13075-020-02281-1

**Published:** 2020-09-21

**Authors:** Yongyao Wu, Xiaomin He, Ning Huang, Jiayun Yu, Bin Shao

**Affiliations:** 1 State Key Laboratory of Oral Diseases, National Clinical Research Center for Oral Diseases, West China Hospital of Stomatology, Sichuan University, Chengdu, 610041 China; 2 State Key Laboratory of Biotherapy anf Cancer Center, West China Hospital, Sichuan University, Chengdu, 610041 China

**Keywords:** A20, Arthritis, Zinc finger domains, Inflammation, Mouse models

## Abstract

A20, also known as TNF-α-induced protein 3 (TNFAIP3), is an anti-inflammatory protein that plays an important part in both immune responses and cell death. Impaired A20 function is associated with several human inflammatory and autoimmune diseases. Although the role of A20 in mediating inflammation has been frequently discussed, its intrinsic link to arthritis awaits further explanation. Here, we review new findings that further demonstrate the molecular mechanisms through which A20 regulates inflammatory arthritis, and we discuss the regulation of A20 by many factors. We conclude by reviewing the latest A20-associated mouse models that have been applied in related research because they reflect the characteristics of arthritis, the study of which will hopefully cast new light on anti-arthritis treatments.

## Introduction

Arthritis has long attracted much attention due to its multifaceted influence on human health; the three most common types are rheumatoid arthritis (RA), osteoarthritis (OA), and spondyloarthritis (SpA) [1]. RA, characterized by painful swollen joints and progressive bone erosion, is an autoimmune disease that severely impairs physical function and quality of life [2]. OA is the primary cause of disability and source of societal expenditure in elderly adults, which has become even more prevalent recently than it has been in preceding decades [3]. What is worse, there have been non-significant differences to patients’ mortality risk and functional disability between a decade ago and now, but patients’ expectations of efficient treatment have increased [4]. Therefore, it is meaningful to explore innovative therapeutic strategies for arthritis.

RA, OA, and SpA can be distinguished based on their diversity in pathogenesis and manifestations, but they demonstrate analogous inflammatory features; however, not all signs of inflammation appear in joints [1]. In addition, many inflammation-related pathways, such as the tumor necrosis factor receptor (TNFR)-induced nuclear factor kappa B (NF-κB) pathway, mitogen-activated protein kinase (MAPK) signaling, and Janus kinase-signal transducer and activator of transcription (JAK-STAT) signaling, are highly associated with these diseases [5–7]. Pro-inflammatory family members, which can be detected in the articular environment when arthritis occurs, are associated with the degree of inflammation present [8]. On the one hand, some pro-inflammatory cytokines, such as tumor necrosis factor-α (TNF-α), interleukin (IL)-6, and IL-1, facilitate the development of arthritis [9]. On the other hand, IL-4, IL-10, IL-37, and IL-38 play the role of relieving inflammation to restrict arthritis [10], particularly A20.

**Fig. 1:**
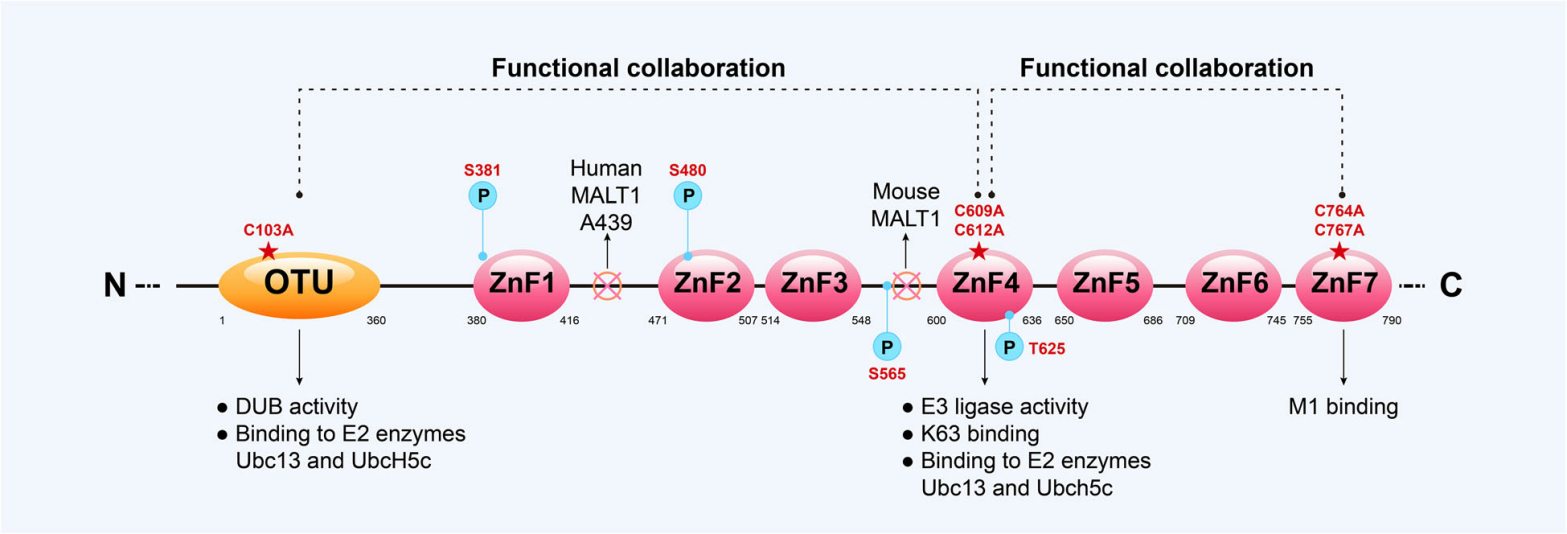
Domain structure and biochemical characteristics of the A20 protein. The typical amino acid sequence of human A20 has been digitally annotated. The A20 protein contains an ovarian tumor (OTU) domain in its amino terminus and seven zinc fingers (ZnF) in its carboxy-terminal end, and each domain has its own characteristics in mediating deubiquitylating (DUB) or ubiquitylating (Ub). The OTU domain of A20 has deubiquitylating enzyme activity, which is mediated by the catalytic residue Cys103 (C103). A20 is capable of binding to ubiquitylated E2 enzymes such as Ubc13 and UbcH5c via OTU and ZnF4. The ZnF4 domain of A20 mediates E3 ubiquitin ligase activity and has K63-linked polyubiquitin-binding affinity. ZnF7 has M1-linked polyubiquitin affinity and competes with other Ub-binding proteins to prevent the degradation of M1-linked chains. A20 also interacts with other protein such as RIP1, TAX1-binding protein 1 (TAX1BP1), and UbcH5a via ZF domain, and with TNF receptor associated factor 6 (TRAF6) via OTU domain. What is more, A20’s ZnF4 and ZnF7 Ub-binding domains are synergistic in regulating Ub-dependent signaling. OTU and ZnF4 domains of A20 complement each other in cells, which is facilitated by dimerization of A20 proteins. A20 can be regulated by posttranscriptional modification. For example, Human A20 is cleaved by MALT1 at Ala439, while mouse A20 is cleaved at the site between ZnF3 and ZnF4. Phosphorylation sites of A20 contains Ser381, Ser480, Ser565, and Thr625, and phosphorylation at Ser381 mediated by IKKβ can improve DUB activity. Additionally, domain-specific mutant mice can be generated by mutating OTU (C103A), ZnF4 (C609A and C612A), or ZnF7 (C764A and C767A) and other specific sites

A20, also known as TNF-α-induced protein 3 (TNFAIP3), was originally discovered in 1990 by Dixit [11]. It is a highly conserved protein that contains an amino-terminal ovarian tumor (OTU) domain in its N-terminus and seven zinc finger (ZnF) domains in its C-terminus. The OTU domain mediates deubiquitinating (DUB) activity, and the ZnF4 and ZnF7 domains bind to K63-linked Ub (ubiquitin) chains and M1-linked Ub chains, respectively, and support E3 Ub ligase activity (Fig. 1). Therefore, A20 acts as an “ubiquitin-editing” enzyme because it possesses both deubiquitinating and ubiquitinating abilities [12, 13]. After decades of study, the capabilities of A20 have been revealed to a certain extent. A20 has been well established as a negative regulator of NF-κB in response to TNF, IL-1, IL-17, and so on [12, 14], though there are paradoxical in vitro and in vivo findings about the role of A20 in attenuating inflammatory signaling [15]. Improper activation of NF-κB implicates the development of chronic inflammation and cell death and is thought to be a therapeutic target for arthritis, though it plays a key role in activating the innate and adaptive immune system to protect against infection and promote survival [16, 17]. In these processes, A20 is thought to disrupt the ubiquitination status of specific signaling proteins in the TNFR and Toll-like receptor (TLR) pathways to inhibit NF-κB signaling [18, 19], which occurs via the deubiquitinating activity of the OUT domain and the inverse Ub-binding function of ZnF domains [12]. Gradually, new anti-inflammatory functions, such as regulating the activation of inflammasomes and restricting the secretion of pro-inflammatory cytokines such as interleukin [20], are being identified for A20, further demonstrating its potential roles in anti-inflammation.

The inflammatory features of arthritis and the anti-inflammatory function of A20 suggest that A20 can suppress arthritis by restricting inflammation, which has been verified by a growing number of studies. Evidence for the antiarthritic function of A20 was provided by myeloid-A20-deficient mice, which developed swelling and redness of the front and hind paws that reached 100% at 20 weeks of age, as shown by Matmati et al. [21]. Thus, A20 has established its position as a potential molecule that could affect the course of arthritis. Recent studies have made further progress on understanding A20 and have shown that it protects cells from TNF-induced apoptosis through linear ubiquitin-dependent and linear ubiquitin-independent mechanisms [22, 23]. It has also been confirmed recently that there is a tight association between TNFAIP3 gene polymorphisms and RA susceptibility [21, 24, 25]. In addition, A20 haploinsufficiency (HA20) can cause early-onset inflammatory arthritis in humans [26], which further illustrates the significant role of A20 in arthritis. A recent study even revealed that increased expression of type I interferon (IFN) was correlated with autoinflammatory disease activity in HA20 patients, while A20 was capable of restraining the type I IFN response, though the underlying mechanism needs further exploration [27]. The highlight of these studies is that the ZnF4 and ZnF7 ubiquitin-binding domains in A20 have been proven to be critical to the function of preventing inflammation-dependent arthritis [28–30], and exploration of the specific role of these domains has gradually progressed. For example, several mouse models have proven that the ZnF7 domain of A20 has a particular status in regulating spontaneous immune activation and preventing cell necroptosis; further ZnF4 plays a role in the process, as it has the property of acting synergistically with ZnF7 [28, 29].

In this review, we summarize some antiarthritic mechanisms of A20 based on its anti-inflammatory features and summarize factors affecting A20 expression. We also highlight the review by listing mouse models that can simulate arthritis for the A20 study, which could facilitate research on this promising protein.

## Key molecules implicated in the pathogenesis of arthritis

**Fig. 2:**
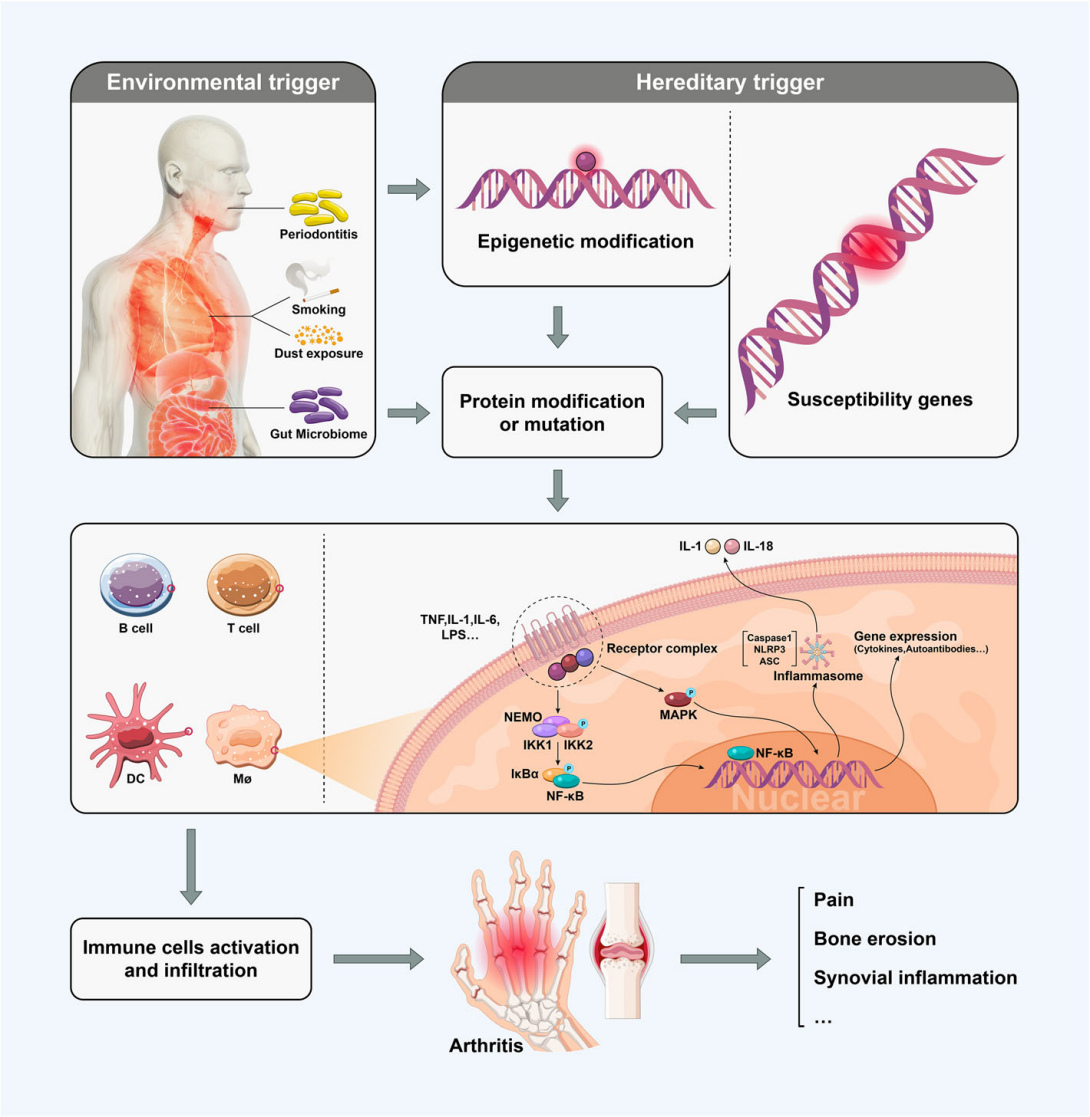
Principle of the development of arthritis. Hereditary and environmental factors synergistically trigger protein mutation or modification. First, the protein mutation results in the activation of immune cells such as macrophages (Møs) and dendritic cells (DCs), leading to hypersensitivity of these cells to TNF, LPS, IL-1, and so on. Second, after modification, the specific peptide of the protein is recognized by DCs, and DCs can also submit abnormal protein fragments to T cells, resulting in the activation of immune cells. Then, NF-κB and MAPK are activated. As a consequence of the activated NF-κB pathway, inflammasomes tend to assemble, pro-inflammatory cytokines and autoantibodies are produced, and immune cells infiltrate. Thereafter, localization of the inflammatory response occurs, and arthritis is initiated

A severe and persistent inflammatory response is one of the hallmarks of arthritis. Previous studies revealed that environmental and hereditary factors trigger arthritis by protein modification, mutation, and immune cell activation [9, 31] (Fig. 2). During this process, cytokines, such as TNF and IL-6, also play a critical role in the pathogenesis of inflammation in arthritis. TNF aggregates peripheral MDSCs, which are ubiquitous in inflammation [32]. Meanwhile, TNF-α could also induce an inflammatory response, which involves the activation of leukocytes, cytokines, and chemokines, stimulating the formation of osteoclasts and promoting the resorption of cartilage and bone [9]. IL-6 also activates leukocytes and osteoclasts and increases autoantibody production, which is closely related to OA [33]. In addition, IL-1β, IL-6, IL-21, and IL-23 support the differentiation of T helper 17 (Th17) cells, suppress the differentiation of regulatory T cells, and cause inflammation of arthritis [9]. IL-17 has powerful properties in inducing neutrophil-attracting chemokines to initiate and promote inflammation and helps to destroy cartilage and increase osteoclast differentiation, leading to bone erosion in joints [9, 34, 35]. Moreover, the sustained activation of the IL-17 axis leads to an accumulation of immune cells in the synovium and increased intercellular interactions, thereby forming a pro-inflammatory environment that could in turn promote the synergistic collaboration of IL-17 with TNF-α, IL-1β, IL-18, and many other cytokines [35]. This further aggravates inflammation.

Moreover, the intracellular signaling pathways related to inflammation in arthritis involve NF-κB, Janus kinase (JAK), MAPK, and so forth (Fig. 2). NF-κB can be independently activated by TNFR-1, Toll-like receptor, IL-1R, and others [36]. In addition, MAPK is considered to be the intersection of multiple signaling pathways and the upstream signal of the NF-κB signaling pathway, as the activated MAPK signaling pathway participates in mediating the activation of NF-κB [9]. However, strategies targeting these pathways might enable therapeutic avenues for relieving the symptoms of arthritis.

Accordingly, the strategy of targeting these cytokines with inhibitors and neutralizing antibodies is widely used in arthritis therapy [10, 31]. TNF-α inhibitors can significantly improve the clinical symptoms of human TNF transgenic (hTNFtg) mice with spontaneous arthritis, and they are frequently used in patients with severe RA [37, 38]. IL-6-receptor antibodies in RA treatment, such as tocilizumab, showed outstanding efficacy [39]. In addition, it has been reported that granulocyte-macrophage colony-stimulating factor (GM-CSF) inhibition is effective in treating RA [40].

## Mechanism of A20 regulating arthritis

### A20 restricts arthritis development by controlling TNF and the interleukin signaling pathway

As mentioned above, TNF-α inhibitors have been frequently used to treat severe RA [38] because of the pioneering status of TNF-α at the apex of the pro-inflammatory cytokine cascade [6, 41]. Moreover, the activation of TNF-induced TNFR signaling and downstream signaling evoked by TNFR, such as NF-κB and MAPK, implicates a variety of responses. For example, activated NF-κB further reinforces inflammation by triggering the formation of numerous pro-inflammatory cytokines, such as TNF-α, IL-1β, and IL-6, and repressing IL-4 [17]. Additionally, when TNFR1 and receptor interacting protein kinase 1 (RIPK1) are modified by lysine-63 (K63)-linked ubiquitin ligases, they can generate a signaling complex that facilitates NF-κB activation [42]. Otherwise, in ablation of *TNFAIP3*/A20, upon TCR and CD28 co-stimulation and with the help of a TNF inhibitor, protein kinase C (PKC) and p38 MARK signaling are in an active state, leading to a dramatic increase in the number of Th17 cells circulating in the blood, and the level of IL-17a correspondingly increases [43]. Another study reported that a tendency towards high levels of type I IFN can be detected during anti-TNF-α therapy, which has been deemed a marker of anti-TNF-α failure. Therefore, it is reasonable to hypothesize that elevated IL-17a, type I IFN, and other cytokines during the use of TNF inhibitors might be the reasons for the failure of these treatments [38].

**Fig. 3:**
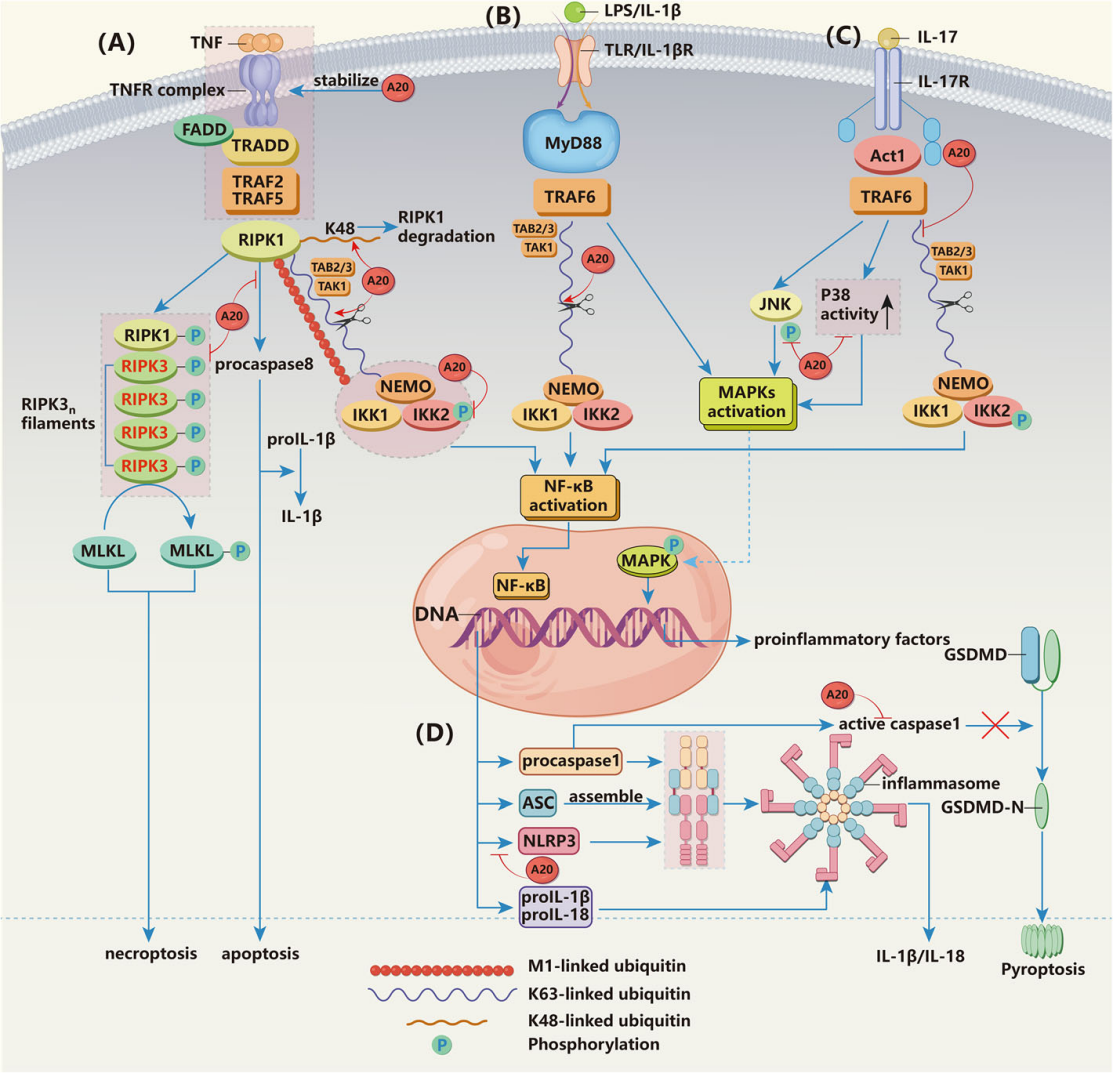
The mechanism by which A20 regulates arthritis. (**a**) In the TNF-induced NF-κB pathway, A20 can impair IKK complex activation, thus opposing the activation of NF-κB. Moreover, A20 removes lysine-63 (K63)-linked ubiquitin chains from RIPK1 and TNFR1 and adds K48-linked ubiquitin to RIPK1, thus targeting RIPK1 for proteasomal degradation and leading to dissociation of a RIPK1-containing complex from the membrane; these processes eventually block NF-κB-mediated promotion of cell survival. Degradation of RIPK1 avoids procaspase 8-dependent apoptosis and procaspase 8-mediated cleavage of pro-IL-1β. Additionally, phosphorylation of RIPK3 in the RIPK3-dependent RIPK1-RIPK3 complex is suppressed when A20 is present, and RIPK1-RIPK3-MLKL-dependent necroptosis is accordingly reduced. Furthermore, A20 stabilizes the connection of the M1-linked ubiquitin chain to complex I through its ZnF7 domain to protect them from being degraded by other DUBs and avoid necroptosis. **b** TLR-induced NF-κB activation due to LPS and IL-1β stimulation can be interrupted by A20 by impairing IKK complex activation as described above. A20 can also remove K63 ubiquitin chains on TRAF6, thereby blocking NF-κB and preventing MAPK activation to reduce IL-17 expression. **c** IL-17-induced activation of NF-κB can be restricted by A20 by removing K63-linked ubiquitin chains from TRAF6 and interrupting IKK complex activation. Moreover, A20 can restrict MAPK activation. On the one hand, A20 hinders the phosphorylation of JNK, thereby restricting MAPK signaling and reducing the production of pro-inflammatory factors. On the other hand, the existence of A20 inhibits p38 MARK and PKC activity to decrease IL-17 levels to hinder further inflammatory responses mediated by IL-17. **d** A20 can reduce the transcription of NLRP3, ASC, procaspase 1, pro-IL-1β, and proIL-18, and this regulation relies on the activation of NF-κB and directly lowers the basal expression of NLRP3 to impair inflammatory activation, thus blocking the secretion of IL-1β and IL-18. A20 also has the ability to inhibit pyroptosis in a mechanism that is dependent on active Casp1, thereby restoring the IL-1β production process

A20 is a ubiquitin-editing enzyme that can edit a variety of ubiquitin chains through corresponding Ub-dependent biochemical motifs. The OTU domain of A20 functions as a DUB that cleaves K63-­linked polyubiquitin chains, and phosphorylation of A20 at Ser381, which is mediated by IKKβ, can improve its DUB activity. ZnF4 can bind to E2 enzymes and then inhibit E3 ligase activity dependent on these enzymes, which ultimately enables the building K48-linked polyubiquitin chains. ZnF7 binds to M1-linked polyubiquitin and competes with other Ub-binding proteins to prevent the degradation of M1-linked chains [12, 13] (Fig. 1). These findings reveal that A20 is involved in the pathological processes described above in the following ways. First, A20 has the capacity to restrict NF-κB activation in response to TNF, IL-1, lipopolysaccharide (LPS), IL-17, and so on by inhibiting the inhibitor of κB (IκB) kinase (IKK) complex through a mechanism that is dependent on recruitment to the nuclear factor-κB essential modulator (NEMO) C-terminus [44] (Fig. 3a-c) (Upon TNF stimulation, the linear ubiquitin chain assembly complex (LUBAC) is recruited to the TNFR complex and then modifies NEMO to facilitate IKK activation, while ZnF7 can bind to both NEMO and LUBAC and prevent the TNF-induced binding of NEMO to LUBAC, which demonstrates the noncatalytic binding function of ZnF7 in attenuating NF-κB activity [45]. Furthermore, A20 removes K63-linked ubiquitin chains from RIPK1 and TNFR1 in a process that is dependent on the catalytic residue Cys103 (C103)-based deubiquitinating activity of the OTU domain, and then A20 adds lysine 48 (K48)-linked ubiquitin on RIPK1 though the E3 Ub ligase activity of ZnF4, thus targeting RIPK1 for proteasomal degradation and leading to dissociation of a RIPK1-containing complex from the membrane in a process that results in reducing TNF-induced and NF-κB-supported survival [6, 19]. Thus, A20 protects cells from TNF-induced and NF-κB-mediated inflammation and arthritis (Fig. 3a). Moreover, during anti-TNF therapy, IL-17 expression is increased, which is due to a tendency for MAPK to be at a highly active state [43]. A20 can block IL-17 production by interrupting the MAPK signaling pathway through abrogating K63 ubiquitination of TNF receptor associated factor 6 (TRAF6) and preventing the prolonged phosphorylation of c-Jun-N-terminal-kinase (JNK), which are both important for MAPK activation [46]. Moreover, it has been substantiated in some experiments that p38 MARK and PKC activity are inhibited when A20 is present [5], thus blocking IL-17 production and inflammatory aggravation mediated by IL-17 during anti-TNF treatment (Fig. 3b-c). More recently, it has been shown that A20 restricts Th17 cell expansion and arthritis through its ZnF7 motif [28].

Therefore, in the TNF-α inflammatory pathway, which is upstream of the inflammatory response, A20 can target RIPK1 for proteasomal degradation, thus protecting cells from NF-κB activation and the subsequent production of pro-inflammatory factors. A20 can block IL-17 production and IL-17-mediated inflammatory aggravation during anti-TNF treatment by preventing the prolonged phosphorylation of JNK and inhibiting p38 MARK and PKC activity, which is dependent on the ZnF7 motif of A20. As a result, the development of arthritis is repressed. However, the DUB activity of A20 in vivo and in vitro experiments is controversial. In vitro studies suggest that A20 restricts NF-κB signals via C103-based DUB activity, while in vivo, DUB activity does not play a role in directly inhibiting NF-κB and is dispensable for the regulation of NF-κB signaling [15]. Interestingly, a recent study showed that A20’s ZnF7 domain probably also directly regulates NF-κB signaling and that it is independent of A20’s C103-based deubiquitinating activity [28]. A mixture of K63-linked and M1-linked Ub chains exists in IKK complexes, which could be recognized by ZnF4 and ZnF7, resulting in the restriction of IKKγ activity. ZnF7 is involved in this process though supporting the E3 ligase activity of ZnF4 on RIPK1 and directly restricting the activity of IKKγ resulting from its noncatalytic binding function [23, 28]. Therefore, it can be assumed that the E3 ligase activity of ZnF4 and the noncatalytic binding function of ZnF7 could compensate for the attenuated deubiquitinating function in vivo to restrict TNF-mediated induction of NF-κB.

### A20 restricts arthritis development by controlling NLRP3 inflammasome activity

Genetic variations in proteins, namely, NLRP3 of the NLRP3 inflammasome complex, have been reported to influence the susceptibility and severity of RA [47]. NLRP3 inflammasomes are a group of cytoplasmic protein complexes that are assembled by intracytoplasmic pattern recognition receptors (PRRs), which are a type of molecular platform that can respond to danger and promote maturation and secretion of IL-1β and IL-18 [48] (Fig. 3d). Priming of the NLRP3 inflammasome is promoted through activation of NF-κB, which is induced by microbial or endogenous molecules, and the NLRP3 inflammasome is activated by secondary signals such as ATP, pore-forming toxins, and viral RNA [49]. Moreover, priming of the inflammasome can further positively regulate NLRP3 activation at the posttranscriptional level [49]. Therefore, when cells are stimulated with dangerous factors, gene transcription is upregulated for inflammasome components, such as NLRP3, caspase-1 (Casp1), and apoptosis-associated speck-like protein containing a CARD (ASC); the upregulation is accompanied by increased expression of inflammasome substrates pro-IL-1β, pro-IL-18, and gasdermin-D (GSDMD), which is followed by the assembly of the inflammasome [50]. The assembly of inflammatory bodies triggers active Casp1-dependent proteolytic cleavage, which causes the cytokine precursors pro-IL-1β and pro-IL-18 to mature and become biologically active, producing IL-1β and IL-18, respectively [51]. IL-1β recruits innate immune cells to the site of infection and mediates cartilage destruction [52], along with IL-18, which promotes cell lysis and interferon-γ (IFN-γ) production to accelerate the development of inflammation [49].

The levels of NLRP3 and pro-IL-1β in A20-deficient cells are higher than those of wild-type macrophages at rest and those that have been primed with LPS [53]. This evidence suggests a strong connection between A20, the inflammasome, and pathogenesis of RA. A20 can limit the inappropriate activity of this inflammasome by restricting its assembly and suppressing activation of Casp1. First, NLPR3 priming is accomplished by NF-κB activation and translocation of NF-κB, which leads to increased synthesis of NLRP3 so that A20 could exert its NF-κB suppressing function to downregulate NLRP3 inflammasome assembly [54]; further, A20 can also lower the basal expression of NLRP3 directly to impair inflammasome activation [20]. Interestingly, A20’s ZnF7 motif cannot prevent spontaneous NLRP3 inflammasome activation, whereas its ZnF4 and ZnF7 motifs synergistically restrict NLRP3 inflammasome activity [28]. Moreover, as described above, activation of the NLRP3 inflammasome accompanies Casp1 maturity, whereas Casp1 helps mature macrophages and excretes cytokines that promote inflammation [51]. Significant Casp1 activity can also cause pyroptosis, subsequently activating the inflammasome to produce a damage-related molecular pattern (DAMP), which directly drives secretion of pro-inflammatory cytokines [49]. A20 has the ability to inhibit pyroptosis through a mechanism that is dependent on Casp1, thereby preventing the maturation of pro-IL-1β, but the exact mechanism remains unknown [20] (Fig. 3d). Additionally, the substrate of Casp8, which derives from the fraction of RIPK1, is cleaved in pro-IL-1β complexes in A20-deficient cells, and matured Casp8 can accelerate the maturity of pro-IL-1β and mediate apoptosis [55]. However, RIPK1 tends to degrade, and the production of Casp8 is blocked when A20 is present [56] (Fig. 3a).

This evidence suggests that A20 suppresses arthritis development by dampening the function of NRLP3 in several ways. First, A20 downregulates the priming (transcription of inflammasome components) of the NLRP3 inflammasome by limiting the activation of NF-κB, and the activation of NLRP3 is correspondingly restricted, causing a low level of priming. Second, A20 represses Casp1-dependent pyroptosis and the maturation of pro-IL-1β to reduce the production of IL-1β and IL-18. Third, A20 decreases the quantity of Casp8 stemming from RIPK1 to reduce Casp8-dependent apoptosis and the production of IL-18.

### A20 prevents arthritis by inhibiting macrophage necroptosis and restricting spontaneous immune activation through its ZnF7

Necroptosis is a modified form of necrosis in which dead cells break up and release intracellular components, triggering an innate immune response that includes IL-1β release and NLRP3 inflammasome activation [29, 57]. TNFR and RIPK1 mediate intramolecular signaling and necroptosis. TNFR1 recruits an early complex composed of TNFR1-associated death domain protein (TRADD) and RIPK1. In the absence of caspase 8 activity, cellular inhibitors of apoptosis (cIAPs), and LUBAC, RIPK1 can recruit RIPK3 and then forms the RIPK1-RIPK3 complex called the necrosome [57]. Later, mixed-lineage kinase domain-like protein (MLKL) is recruited to this complex and phosphorylated by RIPK3, and the formation of active oligomers leads to plasma membrane destabilization and subsequent necroptosis [57, 58].

A20 can protect cells from necroptosis through its E3 ubiquitin ligase and DUB activities. The kinase activity of RIPK1 and RIPK3 is crucial to the formation of necrosomes, and the phosphorylation and ubiquitination status of RIPK3 as well as the presence of K63-linked polyubiquitin chains are indispensable to the RIPK-dependent necroptosis [57, 58]. As mentioned above, A20 can target RIPK1 for proteasomal degradation by replacing its K63-linked ubiquitin chain with the K48-linked ubiquitin chain and then restricting subsequent signaling, which leads to necroptosis [6, 19, 58]. Additionally, A20 also inhibits RIPK3 ubiquitination with K63-linked polyubiquitin chains through its DUB activity. Interestingly, in A20-deficient mouse embryo fibroblasts (MEFs), more RIPK1-associated RIPK3 phosphorylation is observed than that in WT MEFs after TNF-CHX-ZVAD induction [58], suggesting that A20 reduces the phosphorylated RIPK3, which is dependent on the formation of pro-necroptotic RIPK1-RIPK3 complexes, and further inhibits RIPK3-dependent and phosphorylated MLKL-mediated necroptosis. However, there is a research which showed an inverse effect of A20 in regulating RIPK1. It suggested that elevated A20 expression in inflammatory bowel disease enfeebles the deubiquitination of TNFR1-associated RIPK1 and potentiates intestinal epithelial cell death. It may owe to that A20 dimer binds to linear ubiquitin chains via ZnF7 thus preventing RIPK1 degradation and then enhances caspase-8 activation [59]. And this study is consistent with the research we described above, which shows attenuated function of C103-based DUB activity in vivo.

A20 is also capable of protecting cells from necroptosis independent of its E3 ubiquitin ligase and DUB activities. Recent studies have demonstrated that A20 inhibits macrophage necroptosis via its ZnF7 domain [29, 30]. ZnF7 is vital to the emergence of inflammatory arthritis observed in A20^mZnF7/mZnF7^ (knock-in mice expressing A20 with dual cysteine-to-alanine mutations in ZnF7), as mutations in the ZnF7 domain abrogate the recruitment of TNFR1 signal complexes and diminish linear ubiquitin chains linked to these complexes [60]. A20 stabilizes the M1 (methionine 1)-linked ubiquitin chain connection of TNFR1 signal complexes through its ZnF7 domain to protect them from being degraded by DUBs, which cut linear ubiquitin chains. When harboring abnormal mutations in its ZnF7 domain, the A20^mZnF7/mZnF7^ phenotype ultimately develops inflammation [29] (Fig. 3a). These results indicated that the recruitment of A20 to complex I is dependent on ZnF7 and might be related to the inhibition of macrophage necroptosis.

Moreover, the newly generated mouse model A20^ZF7-CC/ZF7-CC^ (knock-in mice expressing A20 with dual cysteine-to-alanine mutations in ZnF7) has been found to have markedly increased memory phenotype T cells, which are capable of sustaining synovitis and promoting osteoclast differentiation in RA patients [28, 61]. In addition, enhanced NF-κB and JNK signaling have been discovered in naïve A20^ZF7-CC/ZF7-CC^ T cells. Interestingly, these activities were mild in A20^OTU/OTU^ (mice with cysteine-to-alanine mutation of A20’s catalytic cysteine C103) and A20^ZF4/ZF4^ (mice with dual cysteine-to-alanine mutations in A20’s ZnF4 domain) [28]. Therefore, the ZnF7 domain of A20 has been found to have a special and significant status in suppressing T cell activation and expansion. Furthermore, A20^ZF7-CC/ZF7-CC^ and A20^ZF4/ZF4^ macrophages exhibited IL-1β secretion that was restricted by the NLRP3 inflammasome after LPS induction. Inversely, A20^ZF4ZF7/ZF4ZF7^ macrophages showed enhanced NLRP3 inflammasome-mediated caspase-1 activation and pyroptosis, and it illustrated the synergistic function of A20’s ZnF4 and ZnF7 motifs in restricting NLRP3 inflammasome activity and resulting in spontaneous pathology [30]. Together, this evidence illustrates the significance of the ZnF7 domain of A20 in restricting spontaneous immune activation.

Thus, the significance of the ZnF7 domain in necroptosis and spontaneous immune activation has been emphasized, bringing the study of A20 to a new place. This inspired and motivated us to explore more functions of this domain (Table 1).

**Table 1:** The target and mechanism of A20 regulating arthritis

Target	Pro-inflammation approach	Effect of A20	Consequence
NF-κB	Trigger the formation of numerous pro-inflammatory cytokines like IFN-γ, TNF-α, IL-1β, IL-6	Restrict its activation	Reduced pro-inflammation cytokines
Casp1	Cell pyroptosis	Lower activation of Casp1 and inhibit pyroptosis dependent on Casp1	Cell survival and secretion of cytokines is blocked
	NLRP3 activation, IL-1 and IL-18 secretion	Downregulate the transcription of NLRP3, ASC, procaspase1, proIL-1, and IL-18	Inhibited NLRP3 assembly and activation
RIPK1	Activation of NF-κB	Remove K63-linked ubiquitin chains from RIPK1 and add K48-linked ubiquitin on RIPK1	Degradation of RIPK1
	Substrate of Casp8		
MAPKs	Secretion of IL-17A	Deubiquitination of TRAF6 and prevent the prolonged phosphorylation of JNK	Blocked IL-17 production
Unphosphorylated RIPK3	Macrophages necroptosis	Reduce phosphorylation of RIPK3	Macrophages survived
TNFR1 signal complexes	Macrophages necroptosis	Protect these complexes from being degraded through ZnF7 domain	Macrophages survived

## Mechanism of regulation of A20

### TNF-α and interleukin

As its name implies, A20 is notably regulated by TNF-α. TNF-α, IL-1β, IL-17, and other regulators of NF-κB can contribute to the expression of A20 at the transcriptional level by inducing binding to NF-κB binding sites in the A20 promoter [36, 46, 62]. Additionally, TNF-α also increases nuclear expression of GSK3β, thus promoting sustained expression of A20 and terminating NF-κB activity [63]. Meanwhile, A20 not only suppresses NF-κB activity, contributing to reduced production of TNF-α, IL-1β, IL-6, and other cytokines [19, 44], but also regulates IL-1β and IL-17 through TRAF6 and MAPK [5, 46]. As a result, a negative feedback regulation mechanism is formed.

### Adiponectin

Adiponectin, which is secreted mainly by adipocytes, is a vascular protective molecule with insulin-sensitizing, anti-inflammatory, and anti-atherogenic properties [64]. Adiponectin can augment the expression of A20 rapidly and observably by stimulating glucose synthase kinase 3β (GSK3β) activity, resulting in macrophage quiescence and initiating an A20-mediated anti-inflammatory program [65]. Consequently, adiponectin attenuated arthritis severity by suppressing pro-inflammatory markers in a preclinical model of collagen-induced mouse arthritis [66].

### Transcription factors

The downstream regulatory element antagonist modulator (DREAM) is a member of the recoverin subfamily of neuronal calcium sensors, the deficiency of which relieves arthritis inflammatory pain; DREAM is highly expressed in immune cells such as purified CD4^+^ and CD8^+^ T cells [67, 68]. DREAM is a major transcriptional regulator of A20 gene expression, which it achieves through binding to the promoter of the gene encoding A20 [69]. In quiescent states, DREAM is bound to the downstream responsive elements (DREs) to repress A20 transcription in the A20 promoter, which is regulated by Ca^2+^ and interacts with phosphoCREM (cAMP-responsive element modulator). In contrast, upon LPS stimulation, DREAM detaches from DNA to enable the binding of transcription factor upstream stimulatory factor 1 (USF1) to the DRE-associated E-box domain in the gene encoding A20, thereby activating A20 transcription [69]. In general, reduced levels of DREAM protein and activity lead to clearly increased A20 transcription and improved A20-mediated anti-inflammatory response.

Meanwhile, there are other transcription factors regulating the expression of A20, such as orphan nuclear estrogen-related receptor α (ERRα) and Ash1l [70, 71]. ERRα directly binds to the A20 promoter region to promote expression [70], and Ash1l upregulates A20 through H3K4 methylation of the A20 promoter in macrophages [71]. In contrast, methylation of the A20 promoter has also been observed in lymphomas, and it prevents expression of A20 and stimulates NF-κB activation [72].

### microRNA

It has been reported that A20 can be regulated by many microRNAs (miRNAs). The 3′ untranslated region (3′UTR) of the A20 gene was confirmed to be a direct target of miR-125 [73]. MiR-125b was found to be negatively correlated with A20 levels [74], thus strengthening and prolonging NF-κB activity. Furthermore, miR-125b promotes the process of inflammation in RA by activating NF-κB activity [73], and miR-125b is overexpressed in the plasma or serum of RA patients as well [75]. Similarly, miR-19b can also positively regulate NF-κB signaling through synergistic inhibition of A20 [76]. Furthermore, miR-19b is of great importance in the pathology of RA [73], as transfection of miR-19b mimics the increased inflammatory activation of primary fibroblast-like synovial cells in RA [76]. In contrast, although miR-29 has been reported to increase the abundance of A20 mRNA and protein in cancer cells [77], arthritic pathology in miR-29a-deficient mice is reduced [78].

### Others

In addition to the abovementioned regulators, there are other regulators of A20. For example, following TCR stimulation, A20 is degraded by paracaspase MALT1 [79, 80]. In vitro recombinant A20 preferentially hydrolyses K48-linked chains, but phosphorylated A20 cleaves K63-linked chains and enhances ZnF4-mediated substrate ubiquitination [23]. Furthermore, the inhibitory ability of A20 will increase if A20 is phosphorylated at Ser381 by IKKβ [81]. Therefore, we infer that the phosphorylation of A20 is essential for its function. The RNA-binding protein RC3H1 binds the 3′UTR of A20 through ROQ and Zn-finger domains, and knockdown of RC3H1 contributes to increased expression of the A20 protein [82]. In addition, physiological conditions also have a certain effect on A20. Vascular A20 expression can also be regulated by high glucose via O-glucosamine-*N*-acetylation-dependent ubiquitination and proteasomal degradation [83]. Reactive oxygen species promoted A20 expression via increased H3K4me3 modification of histones on the A20 promoter domain [84]. Moreover, by modulating A20 ubiquitylation, RING-type zinc finger protein 114 (RNF114) stabilizes A20 protein [85]. In addition, γ-tocotrienol (γTE) upregulates A20 attributed to increasing phosphorylation of translation initiation factor 2, IκBα, and JNK [86]. Finally, the anti-inflammatory action of gibberellic acid is also thought to significantly increase A20 mRNA and protein levels [87].

## Single nucleotide polymorphisms (SNPs) of the A20 gene and arthritis

Single nucleotide polymorphisms (SNPs) perform a critical function in determining the risk profile of inflammatory and autoimmune diseases. In the A20 gene locus, SNPs are related to inflammatory diseases and autoimmune diseases, including RA [24], psoriasis [88], systemic lupus erythematosus (SLE) [89], and inflammatory bowel disease [90]. The majority of these disease-associated SNPs are located up- or downstream of the A20 noncoding regions or are in intronic regions, suggesting that SNPs in the A20 locus might affect its functional expression. Indeed, two SNPs in the coding region of A20 inducing nonsynonymous mutations (rs5029941/A125 V and rs2230926/F127C) reduce its expression and function [91, 92]. These mutations are both located in exon 3 and have been suggested to affect the DUB activity of A20. In addition to these two coding variants, many SNPs have been identified in noncoding regions and affect the function of cell- and activation-specific enhancers. See details in Catrysse et al. [14], Wertz et al. [23], and Ma et al. [93].

RA has a tight association with several disease-associated SNPs of A20, including rs2230926, rs6920220, and rs10499194 [94, 95]. Studies have established that rs2230926: T > G is strongly associated with RA [94, 96]; this SNP is found to result in a change from phenylalanine to cysteine at position 127, which encodes A20 protein’s deubiquitinase domain and can result in a decreased ability of A20 to suppress NF-κB activation [92]. Associating SNPs in specific genes with arthritis helps to associate genotypes and/or molecular types with prognosis or treatment responses, such as responses to TNF blocked [94, 97]. The Rs2230926 TG genotype and rs146534657 AG genotype have been linked to poor outcomes in RA patients [94]. In addition, patients harboring rs2230926/F127C and rs610604, psoriasis-associated risk genes, respond positively to TNF blocked, so they may be potential prognostic markers [88]. In addition, rs7749323 is a dinucleotide polymorphism of TT > A and can function as an enhancer element binding for NF-κB and the special AT-rich binding protein 1 (SATB1), thereby enabling these proteins to interact with the A20 promoter [98]. Deletion of the TT > A enhancer will result in an enhanced inflammatory response, production of autoantibodies, and inflammatory arthritis [99]. Interestingly, some SNPs in the A20 locus may protect people from RA, such as rs13207033, which is not associated with RA in isolation but is associated in multivariate models [100]. In the absence of rs13207033, the combination of rs6920220 and rs5029937 alleles significantly increased the risk of RA [101]. Rs675520 and rs9376293 were associated with increased joint destruction in ACPA-positive patients but not in APCA-negative patients [102].

HA20 is a rare and early-onset autoinflammatory syndrome due to nonsense and frameshift mutations located in the ZnF4 or the DUB domains of A20 [25]. HA20 patients frequently show increased NF-κB signaling and NLRP3 inflammasome activity, and they develop polyarthritis, which can be the initial disease manifestation [25, 26]. The expression of A20 depends on the topologically associated subdomain (sub-TAD), which contains four enhancers and is important for the functions of regulating the expression of hA20 in vivo and preventing the development of inflammatory pathology [99]. Additionally, the p.(lys91*) a20 mutation can cause hA20, as a result of a severely truncated protein with an impaired OTU domain, which leads to caspase-8-dependent enhanced NLRP3 inflammasome activation and possibly leads to systemic juvenile arthritis and psoriatic arthritis [103]. However, autoinflammatory disease activity in HA20 patients is strongly patient-dependent, ranging from grossly normal to severe multiorgan inflammation, and thus treatment strategies need to vary with each individual [26, 27].

## Mouse models for studying the role of A20 in arthritis

A20-deficient mice exhibit different extents of manifestations that resemble arthritis. Lee et al. first created A20^−/−^ mice that lack A20, but early death of the mice hindered in-depth research [104]. Since then, several A20-associated mouse models have been developed, and they have helped demonstrated the exact relationship between A20 and arthritis; such mouse models include the following: A20^myel-KO^, targeting myeloid cells [21], A20^mZnF7/mZnF7^, targeting the ZnF7 motif, and A20^ZF4ZF7^, targeting ZnF4 and ZnF7 motifs [29]. Here, we summarize their features and how these genotypes imitate typical manifestations of arthritis. Finally, we list some other A20-related models to help study the etiological and pathological role of A20 (Table 2).

**Table 2:** Overview of A20-related mouse models

Genotype	Domain	Alteration	Phenotype	References
A20^−/−^	Full knock out	Full knock out	Severe inflammation, cachexia, and premature deaths	Lee at al. 2000 [104]
A20^myel-KO^	Myeloid-specific	Exon IV and V	Chronic inflammation in ankles, tibiotalar joints and tarsal joints, massive cartilage and bone destruction	Mammati et al. 2011 [21]
A20^mZnF7/mZnF7^	ZnF7	C764A/C767A	Inflammatory arthritis with splenomegaly, symmetrical swelling joints	Polykratis et al. 2019 [29]
A20^ZF7-CC^	ZnF7	C764A/C767A	Psoriasis and psoriatic arthritis	Razani et al. 2020 [28]
A20^ZF4ZF7^	ZnF4 and ZnF7	C609A/C612A	Perinatal lethality, multiorgan inflammation, tissue damage	Razani et al. 2020 [28]
		C764A/C767A		
A20^ZF4ZF7^	ZnF4 and ZnF7	C609A/C612A	Polyarthritis-like manifestations	Razani et al. 2020 [28]
	Myeloid-specific	C764A/C767A		
*Tnfaip3*^OTU^	OTU	C103A	Splenomegaly, ↑IL-1 in response to oral dextran sulfate sodium, ↑IL-6, ↑NF-κB signaling	Lu et al. 2013 [13]
*Tnfaip3*^ZF4^	ZnF4	C609A/C613A		Lu et al. 2013 [13]
A20C103A	OTU	C103A	Grossly normal without inflammation	De et al. 2014 [15]
*Tnfaip3*^*otu/otu*^	OTU	C103A	Sensitive to TNF, LPS, ↑MOG-EAE	Wertz et al. 2015 [23]
*Tnfaip3*^*z4Cys/z4Cys*^	ZnF4	C609A/C612A	Sensitive to TNF, ↑MOG-EAE	Wertz et al. 2015 [23]
*Tnfaip3*^*z4Ub/z4Ub*^	ZnF4	Y599A/F600A	Sensitive to TNF	Wertz et al. 2015 [23]
*Tnfaip3*^1325N^	OTU	I325N	Mild inflammation in pancreatic islets	Zammit et al. 2019 [105]
*Tnfaip3*^C243Y^	OTU	C243Y	Spontaneous inflammatory pathology	Zammit et al. 2019 [105]
*Tnfaip3*^1207L^	OTU	T108A/I207L	Healthy	Zammit et al. 2019 [105]
A20^F^	B cell-specific	Exon IV and V	↑IL-6, ↑B cell proliferation	Chu et al. 2011 [106]
			Chronic inflammation in younger mice and autoimmune syndrome in older mice	
A20^GFP^	CD4 T cell-specific	Exon IV and V	No spontaneous phenotype	Drennan et al. 2016 [107]
			↓invariant NKT cells	
DC ACT	CD11c-specific		↑inflammatory mediators in kidney	Lu et al. 2019 [108]

*OTU* ovarian tumor, *MOG-EAE* myelin oligodendrocyte glycoprotein-induced experimental autoimmune encephalomyelitis, *DC ACT Cd11c*-Cre^+^A20^*flox/wt*^.

### A20^−/−^

To clarify how A20 generally exerts its influence on related inflammation, Lee et al. created A20^−/−^ mouse models in 2000 (Fig. 3) [104]. These A20-deficient mice suffered from severe inflammation and cachexia and died prematurely thereafter. Both unaided and histological observations showed that there was damage and inflammation to different extents in their liver, kidney, intestines, and bone marrow. A20^−/−^ mice were also hypersensitive to low doses of TNF and LPS given that they all died within 2 h of injection [104].

The inability of A20^−/−^ mice to inhibit the NF-κB reaction led to the above symptoms, which are strongly associated with rheumatoid arthritis [104]. It is likely that high levels of TNF-α, secreted by macrophages, induce Th1-mediated inflammation [109]. This is a key cascade in the development of rheumatoid arthritis, causing classic features such as consequent synovial inflammation and joint destruction [110].

### A20^myel-KO^

The A20^myel-KO^ mouse strain, developed by Matmati et al., is deficient in A20 specifically in myeloid cells and demonstrates polyarthritis-like features. Unlike the A20^−/−^ mice described above, A20^myel-KO^ did not suffer from cachexia or prematurely die within 2 weeks, so researchers employ this strain to obtain more data.

The most obvious disparity between A20^myel-KO^ and its wild-type littermates was that these myeloid-deficient mice spontaneously developed polyarthritis in their joints, which closely resembled the histopathological manifestations in humans. For example, A20^myel-KO^ animals suffered from chronic inflammation in their ankles, tibiotalar joints, and tarsal joints, and they also exhibited extensive cartilage and bone destruction [21]. These are all classical manifestations of rheumatoid arthritis. Other abnormal symptoms, such as infiltration of immune cells, erosion, and an increase in blood monocytes and neutrophils, have also been noted [21]. It has also been observed that the serum levels of several arthritis-associated or inflammation-associated cytokines, such as TNF, IL-1β, IL-6, and monocyte chemoattractant protein-1 (MCP-1), were augmented [21]. These cytokines have been proven to be related to the basic pathological process of rheumatoid arthritis. All these autoimmune disorders, regardless of whether they are present in bones, cartilage or blood cells, are hallmarks of rheumatoid arthritis.

Contradicting to initial postulations, the development of polyarthritis in A20^myel-KO^ mice was not dependent on TNF, T lymphocytes, or B lymphocytes, which are involved in the pathogenesis of rheumatoid arthritis [50]. Instead, it was highly associated with TLR4-MyD88-induced signaling and IL-6 [21].

### A20^mZnF7/mZnF7^

The A20^mZnF7/mZnF7^ strain could be potential in studying human psoriasis and psoriasis arthritis, especially those psoriasis patients with anti-cyclic citrullinated peptide (anti-CCP) antibodies, a type of antibody that exists in rheumatoid arthritis. This mouse strain was initially designed to dissect the functional domains in A20 and elucidate their effects on arthritis. Before the advent of this model, evidence has indicated that the ZnF7 domain was capable of binding to linear ubiquitin chains to regulate the TNFR1 signaling pathway, which initiates apoptosis and the necroptosis caspase cascade, thereby leading to inflammatory responses [22, 111]. The same strategic cysteine-to-alanine mutation at 764 and 767 was applied by Polykratis et al. and Razani et al., except that this genotype was respectively referred to as A20^mZnF7/mZnF7^ and A20^ZF7-CC^ [28, 29].

In contrast to A20^−/−^ mice, A20^mZnF7/mZnF7^ mice did not die prematurely, but they weighed less than control mice. A20^mZnF7/mZnF7^ developed inflammatory arthritis with splenomegaly, and researchers observed symmetrical swelling in their ankles, wrists, and toes [29]. Computed tomographic results of their paws revealed bone erosion and reformulation of bone, but compared with symptoms in A20^myel-KO^ [21], the symptoms in A20^mZnF7/mZnF7^ animals were less severe. A20^mZnF7/mZnF7^ also showed higher serum concentrations of two inflammatory cytokines, TNF and IL-6 [30]. A20^ZF7-CC^, the same genotype but with a different name, displayed typical manifestations of psoriasis and psoriatic arthritis, including dactylitis-like erosion in their distal phalanx and nails, enthesitis, aberrant bone formation, erosion, and dermal inflammation [28]. Point mutations in neither OTU nor ZF4 displayed these psoriatic arthritis-like manifestations [28]. Additionally, A20^ZF7-CC/ZF7-CC^ exhibited increased numbers of CD4+ and CD8+ T cells along with an increased proportion of memory phenotype T cells relative to A20^OTU/OTU^, A20^ZF4/ZF4^, and their wild-type littermates [28]. In a similar comparison, the NF-κB and JNK pathways were found to be more active in naïve A20^ZF7-CC/ZF7-CC^ T cells, indicating that the arthritis-like symptoms are T cell-dependent [28]. Intriguingly, A20^ZF7-CC/ZF7-CC^ mice showed elevated levels of anti-CCP antibodies, which is more prevalent in human rheumatoid arthritis than it is in psoriatic arthritis [28]. Hence, this mouse model may constitute a model of synchronized rheumatoid arthritis and psoriatic arthritis.

### A20^ZF4ZF7/ZF4ZF7^

The fact that A20^ZF7/ZF7^ mice far outlives A20^−/−^ animals [28] implies that the role of A20 in regulating inflammation and cell death requires mutual effort of ZnF7 and other domains. Razani et al. generated an A20^ZF4ZF7/ZF4ZF7^ model that harbors point mutations in its ZnF4 and ZnF7 motifs using CRISPR-directed gene targeting [28]. They also generated a tissue-specific model that imitates polyarthritis. A20^ZF4ZF7/ZF4ZF7^ mice exhibited perinatal lethality and multiorgan inflammation similar to what was observed in A20^−/−^ mice [104], and they showed gross inflammation and tissue damage, which was not observed in A20^OTU/OTU^, A20^ZF4/ZF4^, or A20^ZF7-CC/ZF7-CC^ mice [28]. Upon TNF stimulation, A20^ZF4ZF7/ZF4ZF7^ MEFs expressed elevated mRNA that is dependent on NF-κB [28]. Consequent signaling research revealed that A20^ZF4ZF7/ZF4ZF7^ displayed increased phosphorylated IKKβ, expression of phosphorylated IκBα and IKK activity [28].

With the help of the Cre/*loxP* site-specific recombination system, tissue-specific A20^ZF4ZF7/ZF4ZF7^ models have also been put to use. It is worth mentioning that myeloid-specific A20^ZnF4ZnF7/ZnF4ZnF7^ mice exhibit histological and pathophysiological features with polyarthritis, including immune cell filtration, cartilage destruction, and bone erosion [30]. The mice also demonstrated abundant inflammatory cytokines such as TNF and IL-6 in their serum, which was consistent with the A20^myel-KO^ model [30].

### Others

There have been controversies over in vivo studies. For example, *Tnfaip3*^OTU^ data contradicts A20C103A data. *Tnfaip3*^OTU^ mice, with impeded DUB activity, and *Tnfaip3*^ZF4^, with abolished E3 ligase activity, were generated to delineate the role of DUB activity and ZnF4 in restricting TNF signaling. In this study, both *Tnfaip3*^OTU^ and *Tnfaip3*^ZF4^ displayed higher levels of IL-1 in response to oral dextran sulfate sodium, higher levels of IL-6, and increased NF-κB signaling [13]. However, harboring the same mutation as *Tnfaip3*^OTU^, A20C103A mice were grossly normal and exhibited no inflammation compared with heterozygotes and WT littermates [15]. A reasonable explanation for this could be that the DUB activity of A20 collaborates with ZnF4, ZnF7, or other DUBs, such as cylindromatosis [15, 112]. This calls for more mouse models to enable greater depths of study.

As mentioned above, A20 is modified by phosphorylation. Ingrid et al. demonstrated that A20 phosphorylation leads to enhanced K63-linked polyubiquitin chain cleavage performed by the OTU domain, while ZnF4 acts as an accelerator of substrate ubiquitination in vivo through *Tnfaip3*^*otu/otu*^ *Tnfaip3*^*z4Cys/z4Cys*^ and *Tnfaip3*^*z4Ub/z4Ub*^ [23]. Furthermore, three *TNFAIP3 alleles*, *Tnfaip3*^1325N^, *Tnfaip3*^C243Y^, and *Tnfaip3*^1207L^, bearing missense alterations of different extents in the OTU region, showed diminished A20 phosphorylation and elevated immune responses [105]. In comparison, *Tnfaip3*^C243Y^ developed spontaneous inflammatory pathology, while *Tnfaip3*^1325N^ only showed mild inflammation in pancreatic islets, and *Tnfaip3*^1207L^ appeared healthy [105]. Based on these results, Zammit et al. proposed that aberrant mutations in the OTU region modulate A20 phosphorylation and that there might be a tradeoff between impeded A20 activity and microbial resistance [105].

In addition to A20^myel-KO^ mice, B cell-specific knockout A20^F^ mice exhibited augmented IL-6 levels and promoted B cell proliferation and cell survival. Younger mice developed chronic inflammation featuring increased myeloid cells, Tregs and effector T cells, while older mice developed autoimmune syndrome [106]. In CD4 T cell-specific A20 knockout mice, A20^GFP^, there was no spontaneous phenotype. However, A20 deficiency strongly reduced the number of invariant natural killer T (NKT) cells [107]. Further results supported the role of A20 in the proliferation of NKT cells, especially NKT1 and NKT2 [107]. *Cd11c*-Cre^+^A20^*flox/wt*^ mice lack A20 in CD11c-expressing myeloid cells. They displayed elevated inflammatory mediators in their kidneys, and they are more susceptible to inflammatory bowel disease [108].

## Conclusion

After years of exploration in the treatment of arthritis, there is still a lack of an efficient treatment, although TNF inhibitors have been widely used. Based on these anti-inflammatory mechanisms of A20 and evidence of its capacity for arthritic inhibition discussed above, we suggest that A20 is a potential target for arthritis therapies, with the advantages of its multipath effect on arthritis pathogenesis. Therefore, we could design small molecules or genetic techniques to target A20, thus exerting an influence on the expression of A20 to regulate arthritis. Moreover, the A20 rs2230926 TG and rs146534657 AG genotypes may be linked to poor outcome in RA patients. Hence, we could assess these genes to predict the risk of arthritis and sequentially make some precautionary measures to retard the development of arthritis.

Moreover, research on A20 is still far from finished, not only concerning its ability but also regarding its functional domains. The appearance of mouse models A20^mZnF7/mZnF7^ and A20^ZF4ZF7/ZF4ZF7^ inspired us to develop more types of models to probe the function of other domains and the potent synergy between different motifs, thus achieving a complete understanding of A20 and its related diseases.

## Data Availability

All data used to support the findings of this study are included within the article.

## Abbreviations

3′UTR: 3′ Untranslated region
Anti-CCP: Anti-cyclic citrullinated peptide
ASC: Apoptosis-associated speck-like protein containing a CARD
cIAPs: Cellular inhibitors of apoptosis
Casp1: Caspase-1
CREM: cAMP-responsive element modulator
DAMP: Damage-related molecular pattern
DCs: Dendritic cells
DREAM: Downstream regulatory element antagonist modulator
DREs: Downstream responsive elements
DUBs: Deubiquitinating enzymes
ERRα: Estrogen-related receptor α
GM-CSF: Granulocyte-macrophage colony-stimulating factor
GSDMD: Gasdermin-D
GSK3β: Glucose synthase kinase 3β
IFN-γ: Interferon-γ
IKK: IκB kinase
IL: Interleukin
IκB: Inhibitor of κB
JAK: Janus kinase
STAT: Signal transducer and activator of transcription
JNK: c-Jun-N-terminal-kinase
K48: Lysine-48
K63: Lysine-63
LUBAC: Linear ubiquitin chain assembly complex
LPS: Lipopolysaccharide
M1: Methionine 1
MAPK: Mitogen-activated protein kinase
MCP-1: monocyte chemoattractant protein-1
MEF: Mouse embryo fibroblast
miRNA: MicroRNA
MLKL: Mixed-lineage kinase domain-like pseudokinase
Mø: Macrophage
NEMO: Nuclear factor-κB essential modulator
NF-κB: Nuclear factor kappa B
NKT: Natural killer T cell
NLRP3: NOD (nucleotide oligomerization domain)-, LRR (leucine-rich repeat)-, and PYD (pyrin domain)-containing protein 3
OA: Osteoarthritis
OTU: Ovarian tumor
PKC: Protein kinase C
PRRs: Pattern recognition receptors
RA: Rheumatoid arthritis
RIPK1: Receptor interacting protein kinase 1
RNF114: RING-type zinc finger protein 114
SATB1: Special AT-rich binding protein 1
SNP: Single nucleotide polymorphism
SpA: Spondyloarthritis
TRADD: TNFR1-associated death domain protein
Th17: T helper cell 17
TLR: Toll-like receptor
TNFAIP3: TNF-α-induced protein 3
TNFR: Tumor necrosis factor receptor
TNF-α: Tumor necrosis factor-α
TRAF6: TNF receptor associated factor 6
USF1: Upstream stimulatory factor 1
ZnF: Zinc finger
γTE: γ-Tocotrienol
